# Mechanism Study of the Protective Effects of Sodium Tanshinone IIA Sulfonate Against Atorvastatin-Induced Cerebral Hemorrhage in Zebrafish: Transcriptome Analysis

**DOI:** 10.3389/fphar.2020.551745

**Published:** 2020-10-02

**Authors:** Zhong-Yan Zhou, Wai-Rong Zhao, Ying Xiao, Jing Zhang, Jing-Yi Tang, Simon Ming-Yuen Lee

**Affiliations:** ^1^Department of Cardiovascular Research Laboratory, Longhua Hospital, Shanghai University of Traditional Chinese Medicine, Shanghai, China; ^2^State Key Laboratory of Quality Research in Chinese Medicine and Institute of Chinese Medical Sciences, University of Macau, Macao, China

**Keywords:** sodium tanshinone IIA sulfonate, cerebral hemorrhage, atorvastatin, hypoxia-inducible factor 1, hemoglobin, carbonic anhydrase, Na+/H+ exchanger, traditional Chinese medicine

## Abstract

Hemorrhage stroke is a severe vascular disease of the brain with a high mortality rate in humans. *Salvia miltiorrhiza* Bunge (Danshen) is a well-known Chinese Materia Medica for treating cerebral vascular and cardiovascular diseases in traditional Chinese medicine. Sodium tanshinone IIA sulfonate (STS) is a water-soluble derivative of tanshinone IIA, which is the main active ingredient of Danshen. In our previous study, we established a zebrafish model of cerebral hemorrhage and found that STS dramatically decreased both the hemorrhage rate and hemorrhage area, although the underlying mechanism was not fully elucidated. We conducted a transcriptome analysis of the protective effect of STS against atorvastatin (Ator)-induced cerebral hemorrhage in zebrafish using RNA-seq technology. RNA-seq revealed 207 DEGs between the Ator-treated group and control group; the expression levels of 53 DEGs between the Ator-treated group and control group were reversed between the STS + Ator-treated group and Ator-treated group. GO enrichment analysis indicated that these 53 DEGs encode proteins with roles in hemoglobin complexes, oxygen carrier activity and oxygen binding, etc. KEGG analysis suggested that these 53 DEGs were most enriched in three items, namely, porphyrin and chlorophyll metabolism, ferroptosis, and the HIF-1 signaling pathway. The PPI network analysis identified 12 hub genes, and we further verified that Ator elevated the mRNA expression levels of hemoglobin (*hbae1.3*, *hbae3*, *hbae5, hbbe2*, and *hbbe3*), carbonic anhydrase (*cahz)*, HIF-1 (*hif1al2*) and Na+/H+ exchanger (*slc4a1a* and *slc9a1*) genes, while STS significantly suppressed these genes. In addition, we found that pharmacological inhibition of PI3K/Akt, MAPKs, and mTOR signaling pathways by specific inhibitors partially attenuated the protective effect of STS against Ator-induced cerebral hemorrhage in zebrafish, regardless of mTOR inhibition. We concluded that hemoglobin, carbonic anhydrase, Na+/H+ exchanger and HIF-1 genes might be potential biomarkers of Ator-induced cerebral hemorrhage in zebrafish, as well as pharmacological targets of STS. Moreover, HIF-1 and its regulators, i.e., the PI3K/Akt and MAPK signaling pathways, were involved in the protective effect of STS against Ator-induced cerebral hemorrhage. This study also provided evidence of biomarkers involved in hemorrhage stroke and improved understanding of the effects of HMG-COA reductase inhibition on vascular permeability and cerebral hemorrhage.

**Graphical Abstract:**
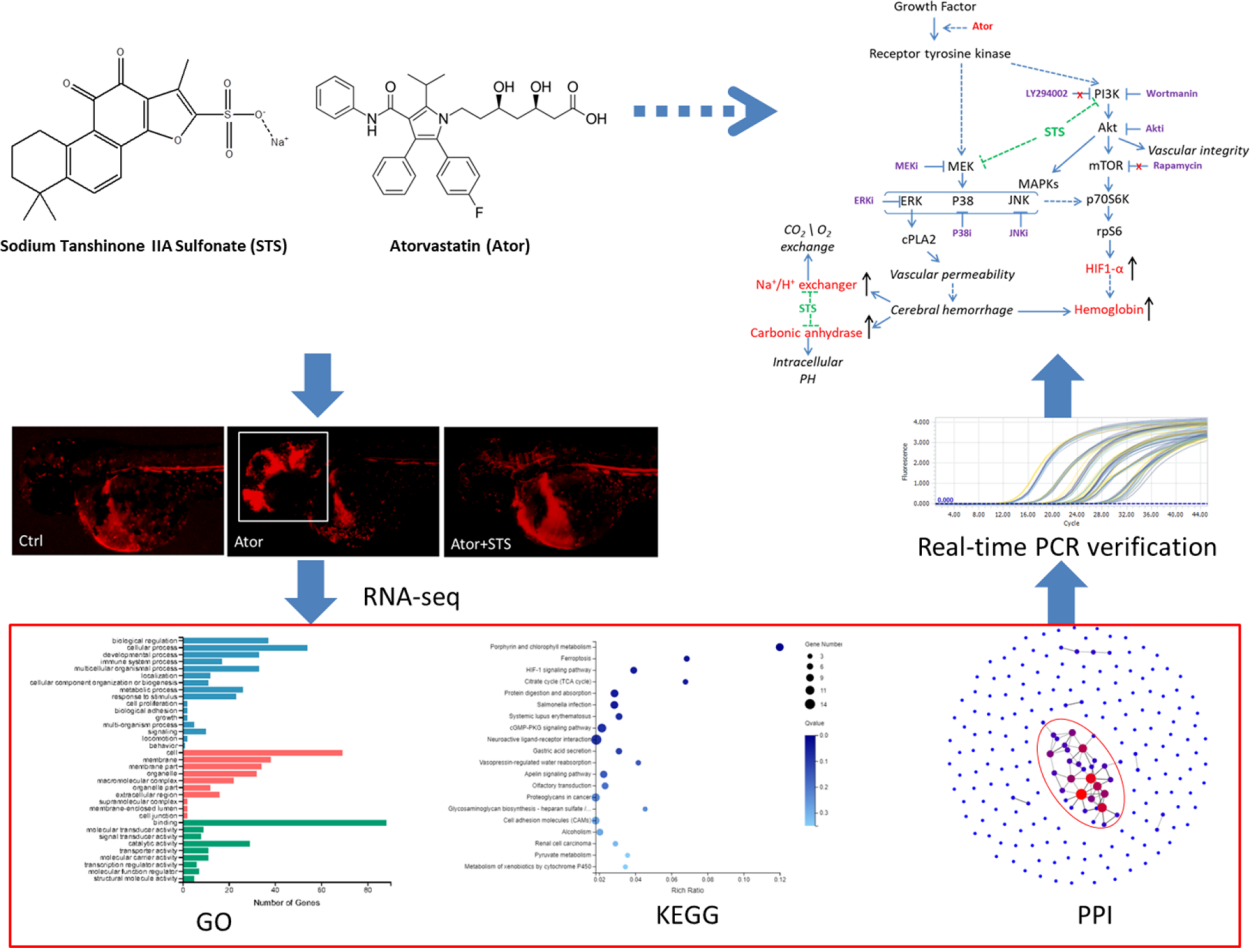


## Introduction

Hemorrhage stroke, which is one of the most severe vascular diseases of the brain, is the second most common type of stroke after ischemic stroke and has a high mortality (Leclerc et al., 2015). Hypertension is the most important risk factor of hemorrhage stroke (Garg and Biller, 2019), although current smoking, as well as other conditions including atherosclerosis and hyperlipidemia, severely damage vascular function and integrity and also increase the morbidity of hemorrhage stroke (An et al., 2017). Disruption of vascular integrity and function is the essential pathological feature of hemorrhage stroke (Yan et al., 2013; Keep et al., 2014). Blood leaked from ruptured vessel causes inflammation, perihematomal edema and neuronal in brain tissue (Aronowski and Zhao, 2011). Hemorrhagic stroke leads to poor functional outcomes in patients who survive, and mortality and brain injury are significantly correlated with the location and volume of the blood bleeding into the brain and the subsequent hematoma growth (Gioia et al., 2015). Drugs with satisfactory therapeutic effects for hemorrhage stroke have not yet been developed. Maintaining vascular function and preventing injury might be important for hemorrhage stroke treatment and prevention.

**Figure 1:**
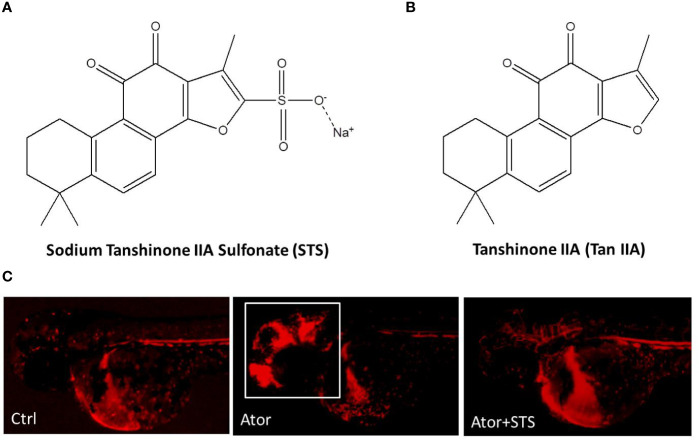
Sodium tanshinone IIA sulfonate (STS) protected against atorvastatin (Ator)-induced cerebral hemorrhage in zebrafish. **(A)** STS, PubChem CID: 23669322. **(B)** Tan IIA, PubChem CID: 164676. **(C)** The transgenic zebrafish line Tg(gata1: dsRed) expressed red fluorescence in blood cells and was employed for observation of Ator-induced cerebral hemorrhage, and the protective effect of STS. One day post-fertilization (1 dpf), zebrafish embryos were treated with STS (100 µM), with or without Ator (2 µM), for 24 h, followed by observation of cerebral hemorrhage under a fluorescence microscope. The white square indicates the region of cerebral hemorrhage in the zebrafish head.

Sodium tanshinone IIA sulfonate (STS) is a water-soluble derivative of tanshinone IIA (Tan IIA), which is the main active component isolated from Chinese Materia Medica *Salvia miltiorrhiza* Bunge (Wang, 2010) (**Figures 1A, B**). STS injection has been approved by the China State Food and Drug Administration (CFDA) for the treatment of cardiovascular diseases, including coronary heart disease, myocardial infarction, and angina (Yang et al., 2009). Recent studies indicated that STS exerts multiple pharmacological activities and could also have the potential for management of ischemia stroke, cardiac hypertrophy, and pulmonary hypertension (Takahashi et al., 2002; Wang et al., 2013; Ji et al., 2017; Zhou et al., 2019c). In our previous study (Zhou et al., 2018), STS significantly decreased both the hemorrhage rate and area in zebrafish in a dose-dependent manner, while STS did not cause toxicity up in zebrafish to 300 µM (**Figure 1C**). STS ameliorated locomotion dysfunction in cerebral hemorrhage zebrafish. The underlying mechanism of action of STS was associated with the maintenance of vascular integrity and cytoskeleton remolding. However, potential biomarkers of Ator-induced cerebral hemorrhage, and the underlying mechanisms of the hemorrhage stroke-protective effect of STS, were not well elucidated.

Thus, in the present study, we investigated the potential biomarkers and underlying mechanisms of STS against Ator-induced cerebral hemorrhage in zebrafish *via* transcriptome profile analysis and RNA-seq technology.

## Materials and Methods

### Chemicals and Reagents

Atorvastatin (Ator) and 3-aminobenzoate methanesulfonate salt (MS-222) were supplied by Sigma (St Louis, MO, USA). Sodium tanshinone IIA sulfonate (STS) was purchased from Chengdu MUST Biotechnology (Chengdu, China), and the purity is HPLC ≥ 92%. The real-time PCR kits were from Roche Life Science (Mannheim, Germany). LY294002, Wortmannin, U0126, Rapamycin, FR 180204, SP600125, and SB239063 were purchased from Beyotime Technology (Shanghai, China). Akt inhibitor IV was obtained from Calbiochem (Darmstadt, Germany). All the chemicals were dissolved in dimethylsulfoxide (DMSO).

### Zebrafish Maintenance and Embryos Collection

Zebrafish were maintained as described in the 4th edition of The Zebrafish Book: A guide for the laboratory use of zebrafish Danio (Brachydanio) rerio by Monte Westerfield, Institute of Neuroscience, University of Oregon. In brief, the zebrafish were maintained under a 14-h/10-h light dark cycle and standard conditions. The fishes were fed twice a day with brine shrimp and, occasionally, normal tropical fish food. Zebrafish embryos were generated by natural pairwise mating and collected within 1 h. Embryos were raised in culture media and incubated in a petri dish at 28.5°C. The zebrafish embryos were anaesthetized by MS-222.

### Drug Treatment

After 24-h incubation, the embryos were dechorionated with a pair of sharp forceps, and any dead, unfertilized, or abnormal embryos were discarded. Embryos were distributed into six-well plates with 40 embryos per group, depending on the assay. One day post-fertilization embryos (1 dpf) were treated with STS (100 µM), with or without Ator (2 µM), for 24 h. Zebrafish embryos treated with E3 buffer containing 1% DMSO was served as the control group. The Tübingen (TU) strain zebrafish was used for transcriptome analysis, while the transgenic zebrafish line Tg(gata1: dsRed) was used for cerebral hemorrhage observation. For evaluation of the inhibition of the PI3K/Akt, mTOR and MAPK signaling pathways, as a protective effect of STS against cerebral hemorrhage, various concentrations (0.3, 1, and 3 µM) of LY294002, rapamycin, wortmannin, Akt inhibitor IV (Akti), U0126 (MEKi), FR 180204 (ERKi), SP600125 (JNKi), or SB239063 (P38i) were co-treated with Ator (2 µM) and STS (100 µM) for 24 h in zebrafish. Cerebral hemorrhage was observed under a fluorescence microscope and the cerebral hemorrhage rate was calculated.

### RNA Extraction, Library Construction and Sequencing

After drug treatment, the heads of zebrafish embryos were harvested (three samples per group). The total RNA of each sample was extracted using TriPure isolation reagent (Roche). The quality of the RNA was measured by the Agilent 2100 Bioanalyzer with the RNA 6000 Nano Kit (Agilent, Santa Clara, CA, USA) the RNA concentration, RIN value, ratio of 28S to 18S, and fragment length distribution were analyzed. Construction of the sequencing library and RNA sequencing were performed by BGI (Shenzhen, China) using the BGISEQ-500 Platform.

### Reads Mapping and Identification of Differentially Expressed Genes

The SOAPnuke analysis tool (v1.5.2; parameters: -l 15 -q 0.2 -n 0.05) was used to measure the sequence quality and filter low-quality reads, i.e., those having quality scores lower than 20; finally, clean reads were obtained in FASTQ format. HISAT2 (v2.0.4; parameters: -p 8 –phred64 –sensitive -I 1 -X 1000) was employed to map the clean reads to the zebrafish (*Danio rerio*) reference genome NCBI_GRCz11. Then, clean reads were mapped to the reference transcript using Bowtie2 (v2.2.5; parameters: -q –phred64 –sensitive –dpad 0 –gbar 99999999 –mp 1,1 –np 1 –score-min L,0,-0.1 -p 16 -k 200) and the gene expression values were calculated by RSEM (v1.2.12; parameters: default). The differentially expressed genes (DEGs) were detected by DEGseq2 software according to the parameters of fold change ≥ 2 and adjusted p value < 0.05.

### Gene Ontology and Kyoto Encyclopedia of Genes and Genomes Enrichment Analyses of DEGs

The Gene Ontology (GO) annotation results for the DEGS were classified, including in terms of the molecular function, cellular component and biological process. The GO enrichment analysis of DEGs was conducted *via* the phyper R package. The false discovery rate (FDR) was calculated for each GO item and an FDR ≤ 0.01 was defined as significantly enriched. The same method was used to annotate and enrich the Kyoto Encyclopedia of Genes and Genomes (KEGG) metabolic pathways analysis of DEGs.

### Protein-Protein Interaction Network Analysis of DEGs

Cytoscape software and its applications, including DIAMOND (v0.8.310) and STRING (v10), were used to analyze the protein-protein interaction (PPI) network. Genes with scores ≥ 300 were graphed.

### Total RNA Extraction and RT-PCR Analysis

Total RNA was extracted from 40 zebrafish embryo heads in each treatment group using the TriPure Isolation Reagent (Roche). RNA was reverse-transcribed to single-strand cDNA using the cDNA Synthesis System for RT-PCR (Roche), followed by real-time PCR using SYBGREEN PCR Master Mix (Roche) on the Light Cycle 96 Real-Time PCR System (LC96; Roche). The PCR conditions were as follows: 95°C for 30 s, followed by 45 cycles of 95°C for 5 s and 60°C for 10 s. The mRNA expression level of each gene was normalized to the amount of GAPDH, which served as internal control. The sequences of primers used in real-time PCR are listed in **Table S1**. The 2^-ΔΔCt^ relative quantification method was used for the data analysis.

### Statistical Analysis

Data were expressed as mean ± S.E.M. with at least three independent experiments and analyzed by Graph Pad Prism software (version 5.0). Student’s t-test was used to evaluate the significant difference between two groups, and p < 0.05 was considered significant.

## Results

### Transcriptome Profile of STS and Ator-Treated Zebrafish Generated by RNA-Seq

The STS obviously prevented Ator-induced cerebral hemorrhage in zebrafish (**Figure 1C**). To determine the underlying mechanism of the protective effect of STS against Ator-induced cerebral hemorrhage, we isolated mRNA from the control, Ator, and Ator + STS treatment groups, followed by transcriptome analysis using RNA-seq. Approximately 78.05% sequences matched with annotated genes in the databases and 25,858 genes were detected in total; 207 DEGs were subsequently identified between the Ator-treated group and control group according to the criteria of fold change ≥ 2 and p value < 0.05. Ator induced the up-regulation of 61 DEGs and down-regulation of 146 DEGs. Consistent with this, 346 DEGs were identified between the Ator + STS group and Ator group, where the expression levels of 138 genes were upregulated and those of 208 genes were downregulated.

### Identification of Potential Biomarkers of Ator-Induced Cerebral Hemorrhage in Zebrafish

**Figure 2:**
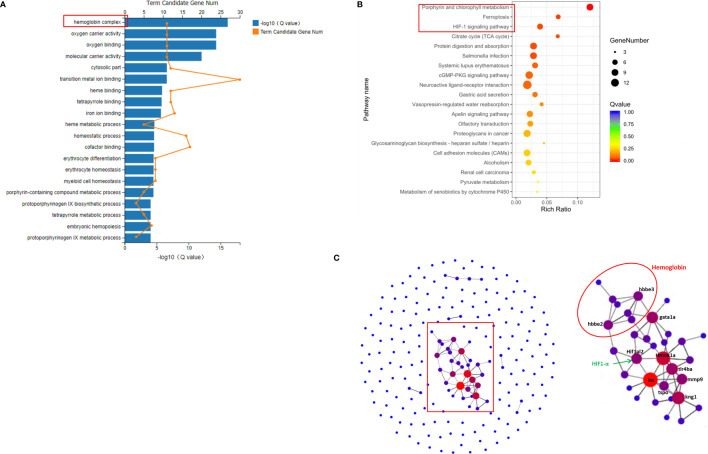
GO, KEGG, and protein-protein interaction (PPI) network analysis of differently expressed genes (DEGs) between the Ator-treated group and Ctrl group. **(A)** GO enrichment of DEGs. **(B)** KEGG pathway enrichment of DEGs. **(C)** PPI maps generated by protein-protein network interaction analysis. The larger node size indicates a higher number of protein interactions.

The 207 DEGs between the Ator-treated group and control group were enriched in 36 different GO terms. The 20 most important terms were selected, several of which were associated with erythrocyte homeostasis, oxygen transportation, and metabolism, such as hemoglobin complex, oxygen carrier activity, and oxygen binding, etc. (**Figure 2A**). In addition, KEGG pathway analysis was used to match these DEGs to physiological processes involved in Ator-induced zebrafish cerebral hemorrhage. Ator treatment affected various signaling pathways, cell metabolism and cell components, including porphyrin and chlorophyll metabolism, ferroptosis, the HIF-1 signaling pathway, the citrate cycle, the cGMP-PKG signaling pathway, cell adhesion molecules (CAMs), etc. (**Figure 2B**). PPI network analysis of the DEGs was used to identify the hub genes most related to Ator-induced zebrafish cerebral hemorrhage (**Figure 2C**). We identified 19 hub genes encoding proteins like hemoglobin and hypoxia-inducible factor 1 α (HIF-1α), etc. based on the criteria of interaction with ≥3 other genes that might be potential biomarkers of Ator-induced cerebral hemorrhage in zebrafish (**Table S2**).

### Identification of Potential Genes Involved in the Protective Effect of STS Against Ator-Induced Cerebral Hemorrhage in Zebrafish

**Figure 3:**
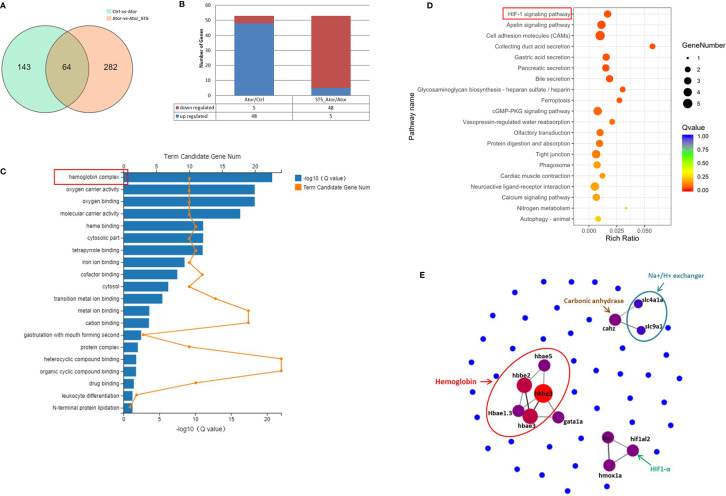
GO, KEGG, and PPI network analysis of DEGs between the Ator+ STS-treated group and Ator-treated group. **(A)** Venn diagram showing the overlap of 63 genes between the Ator-treated group *vs.* control group and Ator + STS-treated group vs. Ator-treated group. **(B)** There were 5 upregulated and 48 downregulated genes between the Ator-treated group vs. control group and Ator + STS-treated group vs. Ator-treated group. **(C)** GO enrichment of DEGs. **(D)** KEGG pathway enrichment of DEGs. **(E)** PPI maps revealed the results of PPI network analysis. The larger node size indicates a higher number of protein interactions.

Regarding potential genes involved in the protective effect of STS against cerebral hemorrhage, we found 64 overlapping genes in the Ator-treated group vs. control group and Ator + STS-treated group vs. Ator-treated group analysis (**Figure 3A**). Furthermore, the expression levels of 53 DEGs, among which five genes were upregulated and 48 genes were downregulated, in the Ator-treated group vs. control group analysis were reversed in the Ator + STS-treated group vs. Ator-treated group analysis (**Figure 3B**). These genes were also subjected to GO and KEGG analysis, the results of which indicated that the mechanisms underlying the protective effect of STS against Ator-induced zebrafish cerebral hemorrhage were involved in hemoglobin complex, oxygen carrier activity, oxygen binding, iron ion binding, CAMs and tight junctions, etc., and their related signaling pathways such as the ferroptosis and HIF-1 signaling pathways, etc. (**Figures 3C, D**). PPI network analysis also indicated that 12 hub genes, which encode proteins like hemoglobin, carbonic anhydrase, Na+/H+ exchanger, HIF-1α, etc., were involved in the protective effect of STS against cerebral hemorrhage and its vascular integrity-promoting effect (**Figure 3E** and **Table S3**). These results indicated that the HIF-1α signaling pathway and physiological processes associated with hemoglobin and oxygen function were most involved in the protective effect of STS against cerebral hemorrhage.

### STS Reduced the Ator-Induced Elevated mRNA Expression Levels of Hemoglobin, Carbonic Anhydrase, and Na+/H+ Exchanger Genes

**Figure 4:**
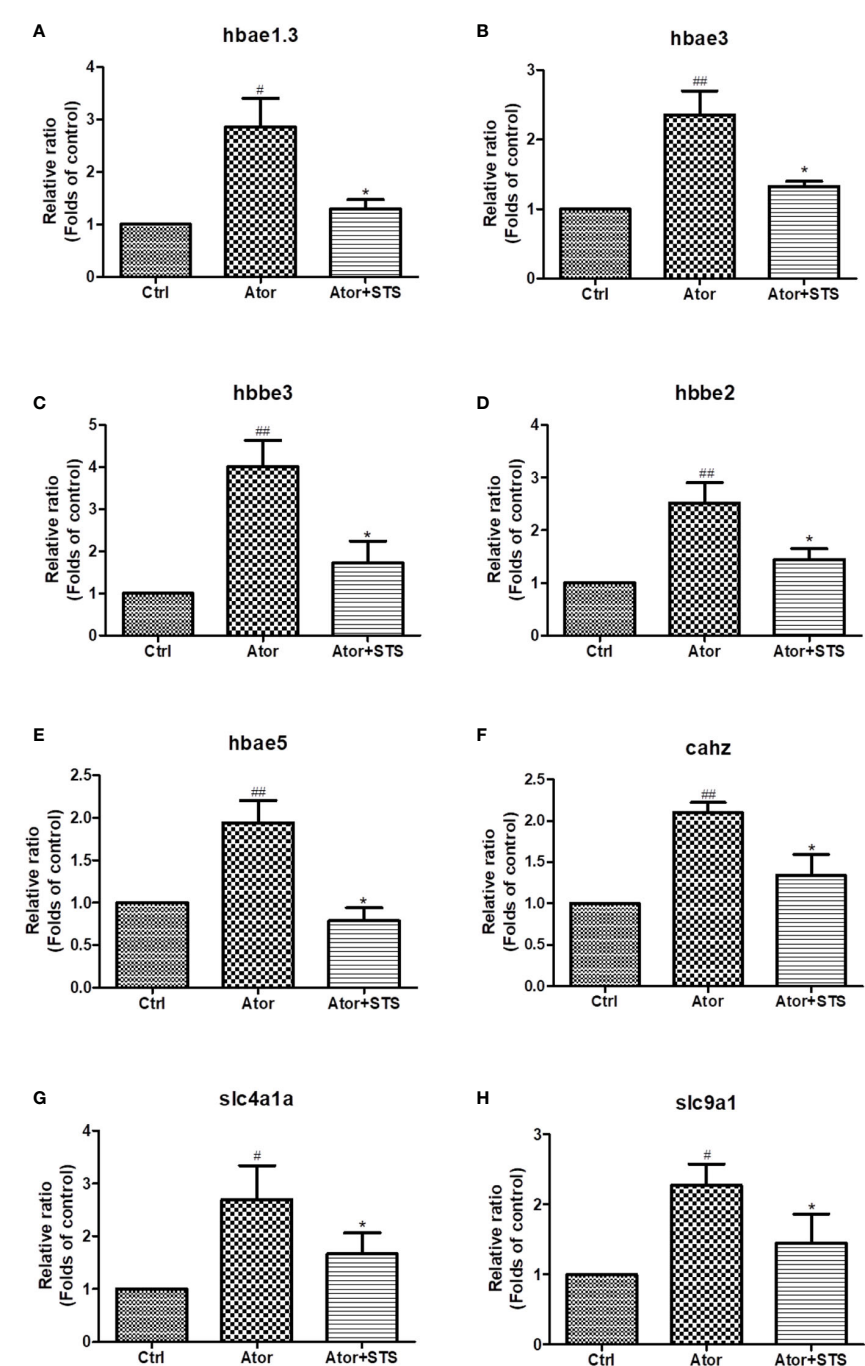
The expression levels of hemoglobin, Na+/H+ exchanger, and carbonic anhydrase genes according to STS and Ator treatment of zebrafish. 1 dpf zebrafish embryos were treated with STS (100µM), with or without Ator (2 µM) for 24 h. The mRNA expression levels of *hbae1.3* **(A)**, *hbae3* **(B)**, *hbbe3* **(C)**, *hbbe2* **(D)**, *hbae5* **(E)**, *cahz* **(F)**, *slc4a1a* **(G)** and *slc9a1* **(H)** genes were detected by real-time PCR. Data are presented as fold changes relative to the control group. ^#^p < 0.05 and ^##^p < 0.01 vs. control group. *p < 0.05 vs. Ator-treated group.

As shown in **Figure 3**, the cellular processes related to oxygen metabolism and exchange were most involved in the protective effect of STS against cerebral hemorrhage. Hemoglobin and carbonic anhydrase are essential for transportation and exchange of CO_2_ and O_2_ in red blood cells (Nikinmaa et al., 2019). Carbonic anhydrase accompanied by Na+/H+ exchange regulates intracellular PH (Parks et al., 2017). The mRNA expression levels of these proteins were further verified by real-time PCR. We demonstrated that the mRNA expression levels of hemoglobin (*hbae1.3*, *hbae3*, *hbae5, hbbe2*, and *hbbe3*), carbonic anhydrase (*cahz*), and Na+/H+ exchanger (*slc4a1a* and *slc9a1*) genes were increased by Ator treatment, and co-treatment of STS with Ator significant decreased the expression levels of these genes (**Figure 4**). Thus, STS dramatically affected oxygen metabolism and exchange in Ator-induced cerebral hemorrhage, and the mRNA expression levels of hemoglobin, Na+/H+ exchanger, and carbonic anhydrase could serve as biomarkers of the efficacy of STS for protecting against cerebral hemorrhage in zebrafish.

### STS Decreased the mRNA Expression Levels of HIF-1, and Its Protective Effect Against Cerebral Hemorrhage Was Partially Blocked by Inhibition of the PI3K/Akt and MAPKs Signaling Pathways Regardless of mTOR Signaling Pathway Inhibition

**Figure 5:**
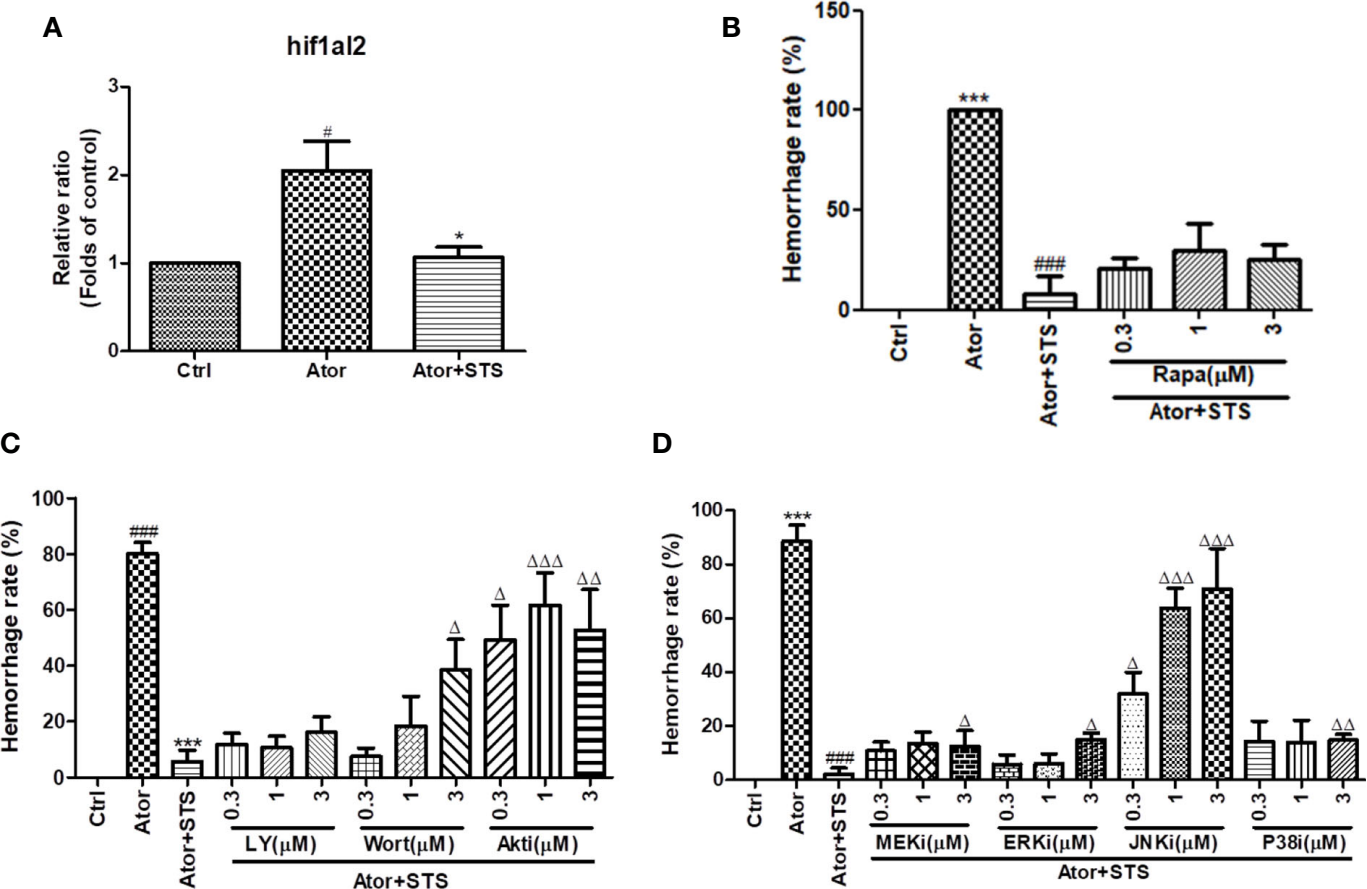
HIF-1 and its related mTOR, PI3K/Akt and MAPK signaling pathways were associated with the protective effect of STS against cerebral hemorrhage. **(A)** Zebrafish embryos (1 dpf) were with treated STS (100 µM), with or without Ator (2 µM), for 24 h. The relative mRNA expression level of the HIF-1 gene *hif1al2* was detected by real-time PCR technology. The results are presented as the fold changes relative to the control group. **(B)** Co-treatment with various concentrations (0.3, 1, and 3 µM) of rapamycin (Rapa) plus Ator (2 µM) and STS (100 µM) for 24 h in zebrafish. **(C)** Co-treatment with various concentrations (0.3, 1, and 3 µM) of LY294002 (LY), wortmannin (Wort), and Akt inhibitor IV (Akti) plus Ator (2 µM) + STS (100 µM) for 24 h in zebrafish. **(D)** Co-treatment with various concentrations (0.3, 1, and 3 µM) of U0126 (MEKi), FR 180204 (ERKi), SP600125 (JNKi), or SB239063 (P38i) plus Ator (2 µM) + STS (100 µM) for 24 h in zebrafish. The cerebral hemorrhage rate was calculated. Data are represented as mean ± SEM. ^#^p < 0.05 and ^###^p < 0.001 versus control group. ***p < 0.001 vs. Ator-treated group. ^Δ^p < 0.05, ^ΔΔ^p < 0.01, and ^ΔΔΔ^p < 0.01 vs. Ator +STS-treated group.

As the GO and KEGG analysis results in **Figure 3D** show, the HIF-1 signaling pathway was the most involved in the protective effect of STS against Ator-induced cerebral hemorrhage. The mRNA expression level of the HIF-1 gene *hif1al2* was elevated by Ator treatment, which might indicate a hypoxia state of cerebral hemorrhage, and co-treatment with STS and Ator decreased its expression (**Figure 5A**). The KEGG pathway analysis revealed that the PI3K/Akt, mTOR and MAPK signaling pathways also co-regulated with the signaling transduction of HIF-1 (**Figure S1**). Thus, we hypothesized that inhibition of the PI3K/Akt, mTOR, and MAPKs signaling pathways might disrupt the protective effect of STS against cerebral hemorrhage in zebrafish. Finally, we found that co-treatment with the mTOR inhibitor rapamycin did not affect the cerebral hemorrhage-protective effect of STS (**Figure 5B**), while PI3K inhibitors (LY294002 and wortmannin), an Akt inhibitor (Akt inhibitor IV), an MEK1/2 inhibitor (U0126), an ERK1/2 inhibitor (FR 180204), a JNK inhibitor (SP600125), and a P38 inhibitor (SB239063) increased the hemorrhage rate to varying degrees (**Figures 5C, D**). These results indicated that inhibition of the PI3K/Akt and MAPK signaling pathways attenuated the protective effect of STS against Ator-induced cerebral hemorrhage in zebrafish. Furthermore, PI3K, Akt, and JNK inhibitors were most effective in suppressing the protective effect of STS against cerebral hemorrhage (**Figures 5C, D**). Thus, these results suggested that STS ameliorated hypoxia in cerebral hemorrhage in zebrafish, and the protective effect of STS against cerebral hemorrhage might also be mediated by HIF-1 and its regulators, including the PI3K/Akt and MAPK signaling pathways.

## Discussion

Hemorrhage stroke is a severe vascular disease of the brain characterized by disruption of blood-brain barrier (BBB) function and of the permeability and integrity of the brain blood vessels required for the normal physiological function of the BBB (Yao, 2019). The effect of HMG-COA reductase inhibitors like Ator on vascular integrity and cerebral hemorrhage still needs further study. STS showed efficacy against cerebral hemorrhage in our previous study, although the underlying mechanism has not been fully elucidated (Zhou et al., 2018). In the present study, we explored the transcriptome profile of Ator-induced cerebral hemorrhage, as well as the protective effect of STS in zebrafish. We summarized DEGs between the control group and Ator-treated group, as well as between the Ator group and Ator + STS group. All of the DEGs were analyzed by GO, KEGG, and PPI network analysis. The mRNA expression levels of genes of interest were verified by real-time PCR, and the possible mechanisms underlying the protective effect of STS against cerebral hemorrhage were also explored by pharmacological inhibition of signaling pathways.

Mice and rat are commonly used in hemorrhage stroke studies. Injection of bacterial collagenase or autologous blood into certain sites in the brain, such as the cisterna magna and striatum, are two main methods to mimic the processes of hemorrhage stroke attack in rodent models (Yagi et al., 2015; Zhang et al., 2015). Zebrafish disease models have the advantages of low cost, ease of observation, and genetic similarity with humans, which facilitates drug discovery (Dooley and Zon, 2000; Kari et al., 2007; Delvecchio et al., 2011). In our group, we have established various zebrafish disease models, including neuroprotection (Chong et al., 2013; Chong et al., 2014), angiogenesis (Zhou et al., 2017; Zhou et al., 2019b; Zhou et al., 2020), cerebral hemorrhage (Huang et al., 2018; Zhou et al., 2019c), etc., for bioactive constitutes discovery from natural products and underlying mechanisms studies. Thus, zebrafish-based cerebral hemorrhage model attracted our attention for high-throughput screening of drugs for hemorrhage stroke (Hung et al., 2012; Crilly et al., 2018). In our previous studies, we established an Ator-induced zebrafish cerebral hemorrhage model, which can also be used to discover agents protecting against cerebral hemorrhage stroke and promoting vascular integrity from traditional Chinese medicine (Hung et al., 2012; Huang et al., 2018; Zhou et al., 2018). STS is a water-soluble derivative of Tan IIA, which is a lipophilic component of Chinese Materia Medica *Salvia miltiorrhiza* Bunge (**Figures 1A, B**). In the present study, we observed a protective effect of STS against Ator-induced cerebral hemorrhage in a transgenic zebrafish line Tg(gata1: dsRed) that expressed red fluorescence in blood cells. Experimental data revealed that STS obviously reversed the Ator-induced cerebral hemorrhage (**Figure 1C**), consistent with our previous study (Zhou et al., 2018).

The hemoglobin and ferric ion released from blood cells caused secondary brain injury by activation of inflammation and oxidative stress in cerebral hemorrhage (Wang et al., 2018; Macdonald and Katan, 2019). Free ferric irons also trigged ferroptosis, which is a novel form of cell death, and caused advanced neuron death (Zhang et al., 2018). Moreover, the elevated ferric ions concentration in brain vascular endothelial cells and pericytes contributed to BBB dysfunction (Imai et al., 2019). GO classification and enrichment analysis revealed that Ator regulated various cellular components and processes associated with erythrocyte homeostasis, oxygen transportation and metabolism, such as hemoglobin complex, oxygen carrier activity and oxygen binding, etc. (**Figure 2A**). In addition, KEGG analysis indicated that Ator treatment affected various signaling pathways, cell metabolism processes, and cell components, including porphyrin and chlorophyll metabolism, ferroptosis, the HIF-1 signaling pathway, the citrate cycle, the cGMP-PKG signaling pathway, CAMs, etc. (**Figure 2B**). Porphyrin and chlorophyll metabolism play vital roles in the formation of hemoglobin, and the cofactor is a well-known porphyrins heme (Senge, 1999). The HIF-1 and cGMP-PKG signaling pathways regulated various physical processes including inflammation, vascular relaxation, and anti-oxidant activity, where these physiological processes are involved in both the development and progression of hemorrhage stroke (Anfossi et al., 2009; Qureshi et al., 2009). The change in citrate cycle-related genes indicated abnormal energy metabolism in hemorrhage stroke. Both CAMs and cell junction also played important roles in vascular integrity and permeability (Zhou et al., 2018; Ziliotto et al., 2019). In our previous study, we found that Ator impaired vascular integrity by disruption of cell-cell adherens junctions (AJs) and STS ameliorated this impairment in vascular endothelial cells (Zhou et al., 2018), which were consistent with the results of KEGG analysis in present study. Finally, PPI network analysis showed that 12 hub genes were most related to Ator-induced cerebral hemorrhage in zebrafish (**Figure 2C** and **Table S2**). These genes encode hemoglobin and HIF-1α etc. and might be potential biomarkers of hemorrhagic stroke.

Moreover, the mechanisms underlying the protective effect of STS against cerebral hemorrhage involved ferroptosis, the HIF-1 signaling pathway, the cGMP-PKG signaling pathway, hemoglobin complex, CAMs, and tight junctions, etc. (**Figures 3A–D**). Twelve highly related DEGs, which encode hemoglobin, carbonic anhydrase, Na+/H+ exchanger, HIF-1α etc. were also been identified between the Ator group and Ator + STS group by PPI network analysis (**Figure 3E** and **Table S3**). These genes might reveal the key mechanisms underlying the protective effect of STS against cerebral hemorrhage in zebrafish. The expression levels of genes of interest, which encode hemoglobin, Na+/H+ exchanger, and carbonic anhydrase, were verified by real-time PCR analysis. Hemoglobin proteins caused dramatic neuronal toxicity in cerebral hemorrhage and regulated the oxygen and carbon dioxide exchange with carbonic anhydrase in blood cells (Hostettler et al., 2019; Nikinmaa et al., 2019). We found that the mRNA expression levels of hemoglobin (*hbae1.3, hbae3, hbae5, hbbe2* and *hbbe3*), Na+/H+ exchanger (*slc4a1a* and *slc9a1*), and carbonic anhydrase (*cahz*) genes were significantly elevated after cerebral hemorrhage, while STS alleviated the expression changes in of these genes (**Figure 4**). Thus, the regulation of oxygen and carbon dioxide metabolism by hemoglobin, Na+/H+ exchanger, and carbonic anhydrase were involved in the mechanism underlying the protective effect of STS against cerebral hemorrhage.

HIF-1α accumulated after cerebral hemorrhage (Jiang et al., 2002; Zhu et al., 2004; Wang et al., 2012); whether HIF-1α is beneficial for cerebral hemorrhage is controversial. We also identified elevated mRNA expression levels of HIF-1 (*hif1al2*) in zebrafish with cerebral hemorrhage, and STS treatment significantly reduced HIF-1 expression (**Figure 5A**). The upregulated expression of HIF-1 indicated hypoxia and insufficient blood supply to the brains of zebrafish, which caused cerebral hemorrhage and neurological deficits. In our previous studies, STS ameliorated cerebral hemorrhage-induced locomotion dysfunction in zebrafish (Zhou et al., 2018). The expression of carbonic anhydrase, which accompanies the Na+/H+ exchanger and contributes to intracellular PH, is also regulated by HIF-1α (Parks et al., 2017). Furthermore, KEGG pathway enrichment analysis suggested that the PI3K/Akt, mTOR, and MAPK signaling pathways might regulate the HIF-1 signaling cascade involved in the protective effect of STS against cerebral hemorrhage (**Figure S1**). PI3K/Akt, mTOR, and MAPK signaling pathways could also trigger normoxic HIF activation (Agani and Jiang, 2013) and regulate cell proliferation and survival, which are essential for maintaining vascular relaxation, angiogenesis, and vascular integrity (Zhou et al., 2017; Liang et al., 2018; Zhou et al., 2019a). The previous study revealed that the activation of Akt and ERK up-regulated the mRNA expression of HIF-1α in brain microvascular endothelial cells (Liang et al., 2018). In addition, the hypoxia-impaired BBB integrity could be reversed by a HIF-α inhibitor (Chen et al., 2019). The PI3K/Akt and MAPK signaling pathways, and HIF-1α, also play critical roles in the prevention of BBB disruption, which is one of the main causes of intracerebral hemorrhage (Zhu et al., 2018). Thus, we hypothesized that the PI3K/Akt, mTOR, and MAPK signaling pathways might be associated with the protective effect of STS against cerebral hemorrhage. As the results in **Figures 5B–D** show, PI3K, Akt, and MAPK inhibitors partially impaired the protective effect of STS against Ator-induced cerebral hemorrhage in zebrafish, regardless of mTOR inhibition. Moreover, PI3K, Akt, and JNK inhibitors were most effective for suppressing the protective effect of STS against cerebral hemorrhage (**Figures 5C, D**). However, the rescue effect of STS along with downregulated *hif1al2* expression was only partially attenuated by Akt and ERK inhibitors (**Figures 5C, D**), in opposite to what is expected based on the results in Liang et al. (2018). STS promoted vascular endothelial function and cell survival through Akt/eNOS and MAPKs signaling pathways (Zhou et al., 2019c). Thus, the activation of Akt/MAPKs signaling pathways might be important in the maintenance of vascular integrity and permeability by STS in cerebral hemorrhage.

**Figure 6:**
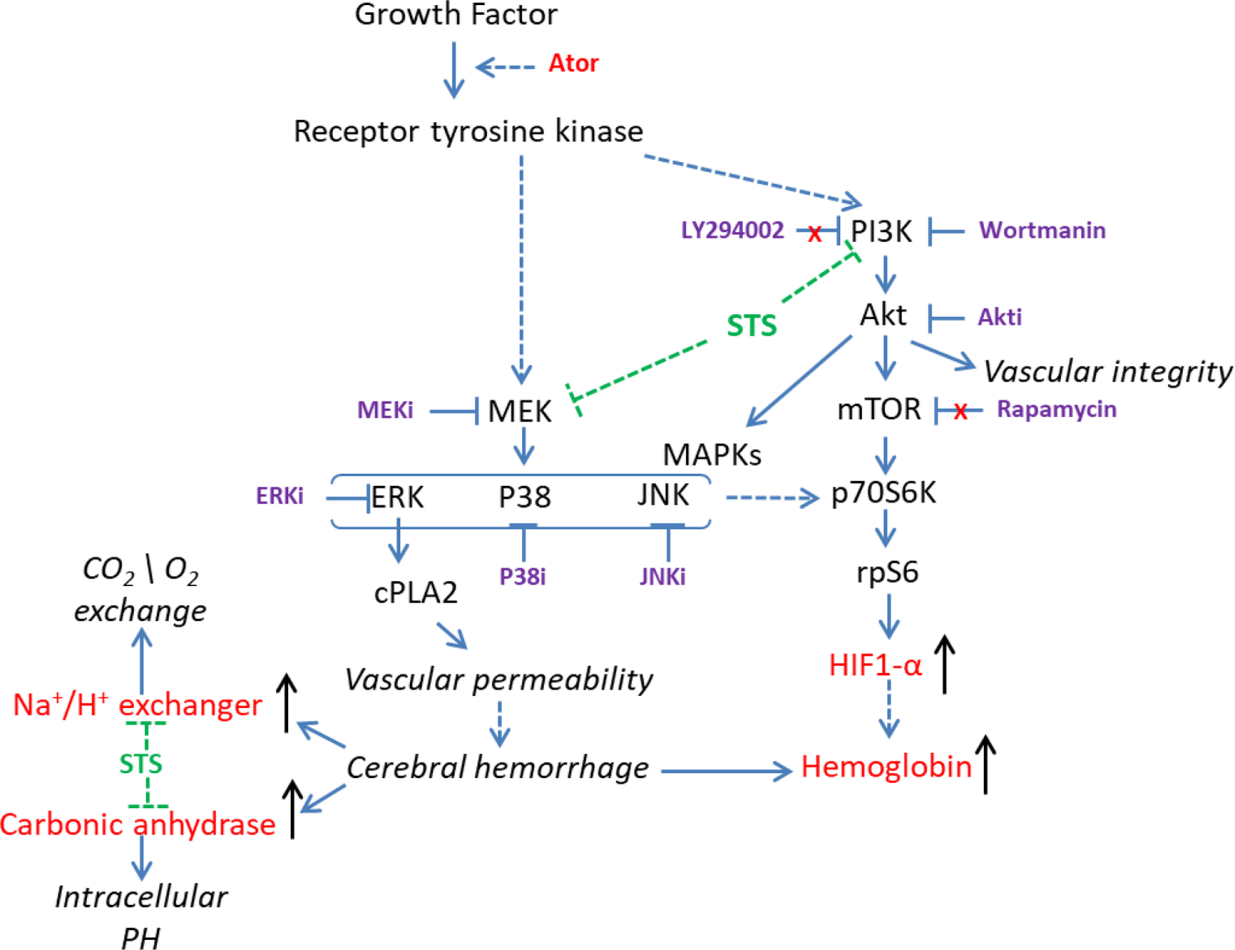
Overview of the possible mechanisms underlying the protective effect of STS against cerebral hemorrhage.

In line with these results, Liu et al. showed that the HIF-1 and PI3K/Akt signaling pathways were involved in hemorrhage stroke by in-depth analysis of the gene expression profile of human cerebral hemorrhage brain samples (Liu et al., 2019). Thus, HIF-1 and its regulators, including the PI3K/Akt and MAPK signaling pathways, might be involved in the protective effect of STS against Ator-induced cerebral hemorrhage in zebrafish (**Figure 6**). In general, the genetic similarity between zebrafish and human is higher than 80%. Due to the presence of physiological difference between zebrafish and human in certain conditions, the research data obtained from zebrafish experiments should be further validated by other animal models. In this study, we had identified the gene expression biomarkers of cerebral hemorrhage and potential pharmacological targets of STS using transcriptome approach. Although the differential gene expression had been verified by independent real-time PCR assay, one main limitation of this approach is the presence of possible discrepancies between mRNA and protein expression regulation. In addition, the direct relationship of the candidate genes could be validated by genetic silencing approaches in future.

In summary, we conclude that the mechanisms underlying the protective effect of STS against Ator-induced cerebral hemorrhage might be associated with HIF-1 and its regulators, including the PI3K/Akt and MAPK signaling pathways. Moreover, hemoglobin (*hbae1.3, hbae3, hbae5, hbbe2*, and *hbbe3*) carbonic anhydrase (*cahz*), HIF-1 (*hif1al2*) and Na+/H+ exchanger (*slc4a1a* and *slc9a1*) genes might be potential biomarkers of Ator-induced cerebral hemorrhage in zebrafish, as well as pharmacological targets of STS.

## Data Availability Statement

The original contributions presented in the study are publicly available. This data can be found here: https://www.ncbi.nlm.nih.gov/sra/PRJNA612371.

## Ethics Statement

All animal experiments were conducted according to the ethical guidelines of Shanghai University of Traditional Chinese Medicine and University of Macau. All experimental protocols were approved by Institute of Chinese Medical Sciences – Animal Ethics Committee (ICMS-AEC) of the University of Macau (IACUC approval number: UMARE-001-2017).

## Author Contributions

Z-YZ, YX, W-RZ, and JZ conducted the experiments. Z-YZ analyzed the data and wrote the manuscript. J-YT and SL designed the study and revised the manuscript. All authors contributed to the article and approved the submitted version.

## Funding

Research at University of Macau was funded by The Science and Technology Development Fund, Macau SAR (File no. 0058/2019/A1), and University of Macau (MYRG2019-00105-ICMS). Research at Shanghai University of traditional Chinese medicine was supported by National Health Commission of Shanghai (GWIV-28, ZY-(2018-2020)-FWTX-8001) and Shanghai University of Traditional Chinese Medicine (A1-U19205010302). Thanks BGI (Shenzhen, China) for RNA-Seq service.

## Conflict of Interest

The authors declare that the research was conducted in the absence of any commercial or financial relationships that could be construed as a potential conflict of interest.

## Abbreviations

Akt, protein kinase B; Ator, atorvastatin; CAM, cell adhesion molecule; CFDA, China State Food and Drug Administration; cGMP, cyclic guanosine monophosphate; Danshen, *salvia miltiorrhiza* Bunge; DMSO, dimethylsulfoxide; DEG, differentially expressed gene; FDR, false discovery rate; GO, Gene Ontology; HIF-1, hypoxia inducible factor-1; HMG-COA, 3-hydroxy-3-methyl glutaryl coenzyme A; KEGG, Kyoto Encyclopedia of Genes and Genomes; MAPK, mitogen-activated protein kinase; mTOR, mammalian target of rapamycin; PI3K, phosphatidylinositol 3-kinase; PKG, cGMP-dependent protein kinase; PPI, protein-protein interaction; RNA-seq, RNA sequencing; Tan IIA, tanshinone IIA; STS, sodium tanshinone IIA sulfonate.
