# Supplementary material for: Mechanism Study of the Protective Effects of Sodium Tanshinone IIA Sulfonate Against Atorvastatin-Induced Cerebral Hemorrhage in Zebrafish: Transcriptome Analysis
Source: Front Pharmacol. 2020 Oct 2;11:551745. doi: 10.3389/fphar.2020.551745 (PMC7567336; doi:10.3389/fphar.2020.551745)
Supplement: Supplementary file 1 [file DataSheet_1.docx]

**Table S1 Specific gene primers used in real-time PCR.**

| **NO.** | **Gene** | **Forward primer** | **Reverse primer** |
| --- | --- | --- | --- |
| 1 | hbae1.3 | 5’- CCTCCAGGCAGCAGACACAG -3’ | 5’- TCCGATGTCGTCAGCCTTTC -3’ |
| 2 | cahz | 5’- TGGAGAAGCTGCCAGTAAGC -3’ | 5’- GATTTGCAGCGCCGATCTTG -3’ |
| 3 | Hif1al2 | 5’- TTGAGTATGGACGGTCAGCG -3’ | 5’- CGACACTCCTGGTGAAGGAC -3’ |
| 4 | hbae3 | 5’- GCACCCAAAGCTGAGGAGAT -3’ | 5’- AGGAGAGTTGGGGCTTAGGT -3’ |
| 5 | hbbe3 | 5’- CACACGCATGAGGAGTGAGT -3’ | 5’- ATTGCCTTCTGAGGGCTGAC -3’ |
| 6 | hbbe2 | 5’- GACCATCGTGATTGCCTCCA -3’ | 5’- GCAGAGACAACAACAGCGAG -3’ |
| 7 | hbae5 | 5’- TGAATTGCACGCCTTTCAGC -3’ | 5’- GCGGTGAAGTCATCAGGGAA -3’ |
| 8 | slc4a1a | 5’- GGCTCAACCAGTTCTCGTCA -3’ | 5’- AACACGGCCAACGATGTACT -3’ |
| 9 | slc9a1 | 5’- TCTGTAAGTGTGTGTGAGATGCC -3’ | 5’- AACTCTCTGATCGGTCTCATTGT -3’ |
| 10 | GAPDH | 5’-GCTTGGCTCCTCTGGCTAAA-3’ | 5’-GTCTTCTGTGTGGCGGTGTA-3’ |

**Table S2 Different expression genes selected on the basis of protein-protein interaction assay (Ator vs Ctrl group).**

| **NO.** | **Gene name** | **Transcripts** | **Nr** | **log2(Ator/Ctrl)** |
| --- | --- | --- | --- | --- |
| **1** | zgc:194125 | NM_001130667.1,XM_021469174.1 | NP_001124139.1\|3.2e-97\|uncharacterized protein LOC100170833 [Danio rerio] | 1.09 |
| **2** | ins | NM_131056.1 | XP_018955433.1\|1.6e-58\|PREDICTED: insulin [Cyprinus carpio] | 1.67 |
| **3** | gata1a | NM_131234.1 | NP_571309.1\|1.7e-243\|GATA binding protein 1a [Danio rerio] | 2.50 |
| **4** | hbbe3 | NM_001015058.1 | NP_001015058.1\|3.1e-78\|hemoglobin beta embryonic-3 [Danio rerio] | 2.14 |
| **5** | hbae3 | NM_183066.2 | NP_898889.2\|2.6e-74\|hemoglobin alpha embryonic-3 [Danio rerio] | 1.82 |
| **6** | epb41b | NM_175084.2 | NP_778259.1\|0.0e+00\|erythrocyte membrane protein band 4.1b [Danio rerio] | 2.34 |
| **7** | tlr4ba | NM_001131051.1,XM_009307228.3 | NP_001124523.1\|0.0e+00\|toll-like receptor 4b, duplicate a [Danio rerio] | 1.19 |
| **8** | hbbe2 | NM_212846.1 | NP_998011.1\|4.1e-79\|hemoglobin beta embryonic-2 [Danio rerio] | 2.38 |
| **9** | mmp9 | NM_213123.1 | NP_998288.1\|0.0e+00\|matrix metalloproteinase-9 precursor [Danio rerio] | -1.28 |
| **10** | egln3 | NM_213310.1,XM_005169814.4 | NP_998475.1\|8.7e-137\|egl nine homolog 3 [Danio rerio] | -1.32 |
| **11** | blvrb | NM_001002686.1 | XP_018977991.1\|4.3e-108\|PREDICTED: flavin reductase (NADPH)-like [Cyprinus carpio] | 1.31 |
| **12** | nt5c2l1 | XM_021479162.1,XM_021479161.1,NM_001004549.2 | NP_001004549.1\|6.5e-307\|5'-nucleotidase, cytosolic II, like 1 [Danio rerio] | 1.73 |
| **13** | kng1 | NM_001005981.1,XM_005170550.4 | NP_001005981.1\|9.0e-194\|kininogen-1 precursor [Danio rerio] | 1.18 |
| **14** | tspo | NM_001006032.2 | NP_001006032.1\|1.5e-92\|translocator protein [Danio rerio] | 1.30 |
| **15** | hif1al2 | XM_005161350.4,XM_021468817.1,NM_001012371.2 | NP_001012371.2\|0.0e+00\|hypoxia-inducible factor 1, alpha subunit, like 2 [Danio rerio] | 2.29 |
| **16** | hbae5 | NM_001326701.1 | NP_001313630.1\|1.4e-75\|hemoglobin zeta [Danio rerio] | 2.95 |
| **17** | hbz | NM_001082834.1 | NP_001076303.1\|8.3e-73\|uncharacterized protein LOC563335 [Danio rerio] | 2.10 |
| **18** | blvra | NM_001076601.1 | NP_001070069.1\|1.5e-163\|biliverdin reductase A [Danio rerio] | 1.21 |
| **19** | hmox1a | NM_001127516.1 | NP_001120988.1\|1.8e-145\|heme oxygenase 1 [Danio rerio] | 2.59 |

**Table S3 Different expression genes selected on the basis of protein-protein interaction assay (STS + Ator vs Ator group).**

| **NO.** | **Gene name** | **Transcripts** | **Nr** | **log2**  **(Ator/Ctrl)** | **log2**  **(Ator_STS/Ator)** |
| --- | --- | --- | --- | --- | --- |
| **1** | ins | NM_131056.1 | XP_018955433.1\|1.6e-58\|PREDICTED: insulin [Cyprinus carpio] | 1.67 | -1.26 |
| **2** | cahz | NM_131110.1 | NP_571185.1\|5.2e-154\|carbonic anhydrase [Danio rerio] | 1.99 | -1.24 |
| **3** | gata1a | NM_131234.1 | NP_571309.1\|1.7e-243\|GATA binding protein 1a [Danio rerio] | 2.50 | -1.87 |
| **4** | hbbe3 | NM_001015058.1 | NP_001015058.1\|3.1e-78\|hemoglobin beta embryonic-3 [Danio rerio] | 2.14 | -1.30 |
| **5** | hbae3 | NM_183066.2 | NP_898889.2\|2.6e-74\|hemoglobin alpha embryonic-3 [Danio rerio] | 1.82 | -1.20 |
| **6** | hbbe2 | NM_212846.1 | NP_998011.1\|4.1e-79\|hemoglobin beta embryonic-2 [Danio rerio] | 2.38 | -1.68 |
| **7** | hif1al2 | XM_005161350.4,XM_021468817.1,NM_001012371.2 | NP_001012371.2\|0.0e+00\|hypoxia-inducible factor 1, alpha subunit, like 2 [Danio rerio] | 2.29 | -1.03 |
| **8** | hbae5 | NM_001326701.1 | NP_001313630.1\|1.4e-75\|hemoglobin zeta [Danio rerio] | 2.95 | -1.49 |
| **9** | hbae1.3 | XM_001333519.7 | NP_891985.1\|4.2e-74\|hemoglobin, alpha embryonic 1 [Danio rerio] | 1.87 | -1.32 |
| **10** | hmox1a | NM_001127516.1 | NP_001120988.1\|1.8e-145\|heme oxygenase 1 [Danio rerio] | 2.59 | -1.50 |
| **11** | slc9a1 | XM_021467239.1,NM_001113480.1,XM_021467241.1,XM_021467240.1 | NP_001106952.1\|0.0e+00\|sodium/hydrogen exchanger 1 precursor [Danio rerio] | 1.66 | -1.17 |
| **12** | slc4a1a | NM_198338.1 |  | 2.28 | -1.16 |


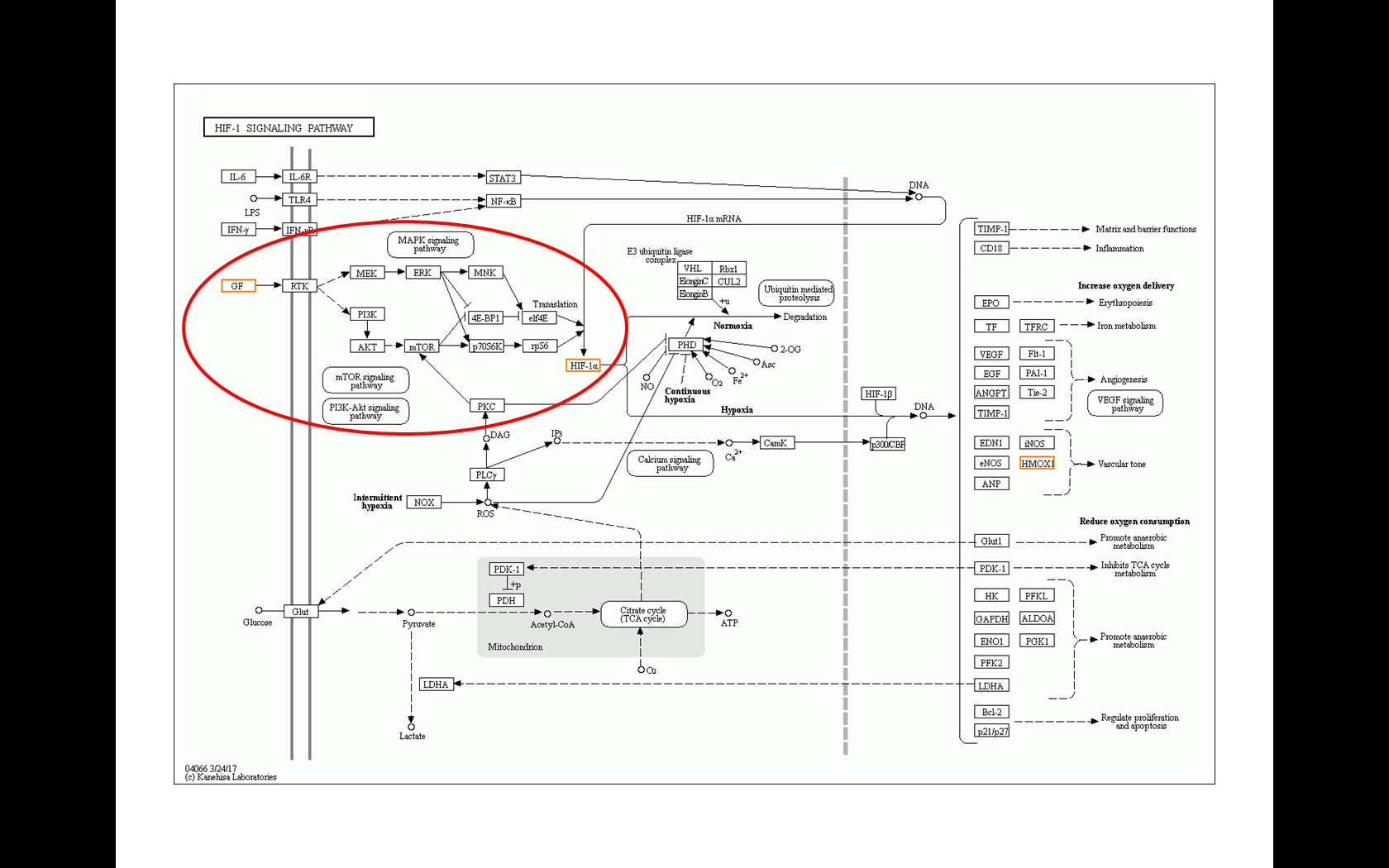


**Fig. S1 HIF-1 signaling pathway was involved in the cerebral hemorrhage protective effect of STS.** Red circle indicated the PI3K/Akt and MAPKs signaling pathways regulate the signal transduction of HIF-1α.
